# Serotonin 5-HTTLPR Genotype Modulates Reactive Visual Scanning of Social and Non-social Affective Stimuli in Young Children

**DOI:** 10.3389/fnbeh.2017.00118

**Published:** 2017-06-23

**Authors:** Antonios I. Christou, Yvonne Wallis, Hayley Bair, Maurice Zeegers, Joseph P. McCleery

**Affiliations:** ^1^School of Applied Social Sciences, Division of Psychology, De Montfort UniversityLeicester, United Kingdom; ^2^Behavioural Brain Sciences Centre, School of Psychology, University of BirminghamBirmingham, United Kingdom; ^3^West Midlands Regional Genetics Laboratory, Birmingham Women’s HospitalBirmingham, United Kingdom; ^4^Department of Complex Genetics, Maastricht UniversityMaastricht, Netherlands; ^5^Center for Autism Research, Children’s Hospital of Philadelphia, PhiladelphiaPA, United States

**Keywords:** 5-HTTLPR, visual scanning, early childhood, non-social cues, aversive stimuli, eye movements

## Abstract

Previous studies have documented the 5-HTTLPR polymorphisms as genetic variants that are involved in serotonin availability and also associated with emotion regulation and facial emotion processing. In particular, neuroimaging and behavioral studies of healthy populations have produced evidence to suggest that carriers of the Short allele exhibit heightened neurophysiological and behavioral reactivity when processing aversive stimuli, particularly in brain regions involved in fear. However, an additional distinction has emerged in the field, which highlights particular types of fearful information, i.e., aversive information which involves a social component versus non-social aversive stimuli. Although processing of each of these stimulus types (social and non-social) is believed to involve a subcortical neural system which includes the amygdala, evidence also suggests that the amygdala itself may be particularly responsive to socially significant environmental information, potentially due to the critical relevance of social information for humans. Examining individual differences in neurotransmitter systems which operate within this subcortical network, and in particular the serotonin system, may be critically informative for furthering our understanding of the neurobiological mechanisms underlying responses to emotional and affective stimuli. In the present study we examine visual scanning patterns in response to both aversive and positive images of a social or non-social nature in relation to 5-HTTLPR genotypes, in 49 children aged 4–7 years. Results indicate that children with at least one Short 5-HTTLPR allele spent less time fixating the threat-related non-social stimuli, compared with participants with two copies of the Long allele. Interestingly, a separate set of analyses suggests that carriers of two copies of the short 5-HTTLPR allele also spent less time fixating both the negative and positive non-social stimuli. Together, these findings support the hypothesis that genetically mediated differences in serotonin availability mediate behavioral responses to different types of emotional stimuli in young children.

## Introduction

Atypical patterns in the visual scanning of images containing negative information or scenes have been widely investigated as a putative marker for the development and manifestation of affective difficulties and disorders (for a review see Boyer and Bergstrom, 2011). In particular, patterns of preferential looking toward and away from threat-related information have been hypothesized to contribute to the development and maintenance of anxiety disorders (e.g., Mathews et al., 1990; Eysenck, 1992; Beck, 2008). For example, an eye-tracking investigation involving negative stimuli presented side-by-side with neutral stimuli for 3 s showed that a young adult population with dysphoria exhibited prolonged viewing of the negative/affective stimuli compared with control participants (Caseras et al., 2007). Similarly, other eye-tracking investigations with longer periods of viewing (e.g., 30 s) different types of emotional versus neutral stimuli have reported that young adults diagnosed with depression exhibit prolonged eye-gaze duration in response to dysphoric images compared with controls (Eizenman et al., 2003; Kellough et al., 2008). However, atypicalities on this type of visual scanning behavior have not yet been firmly associated with a particular psychological construct or psychiatric disorder.

Recently, studies have begun to uncover evidence that atypical visual scanning of negative scenes might reflect a broader and more widespread behavioral trait of over-reactivity, which has also been observed in individuals at increased risk for affective symptomatology (for a review see Ellis et al., 2011). For example, in a behavioral response task it was found that individuals with spider phobia exhibited more rapid visual orienting responses (automatic approach) to spider-related stimuli, but then avoided looking at these negative/affective stimuli compared with a non-anxious control group, reflecting a visual scanning pattern termed *vigilance-avoidance* (Rinck and Becker, 2007). This novel study also highlights an additional distinction which has emerged in the field, i.e., the recognition that aversive stimuli can be either social (e.g., human actions) or non-social (e.g., aggressive animals such as angry dogs and snakes) in nature (for a review see Van Overwalle, 2009; for a recent review on the topic see also Quadflieg and Koldewyn, 2017).

Simultaneous to this recent work, there is research which has highlighted the impact of normal genetic variation in the manifestation of risk for affective traits and disorders. Such investigations, are part of a candidate gene approach which has emerged aiming to investigate the role of common genetic variations that are involved in the neural circuits of emotion regulation and affectivity which may interact with environmental stressors to predict behavioral reactivity, and vulnerability versus resilience for affective disorders (Caspi and Moffitt, 2006; Canli et al., 2006; Canli and Lesch, 2007; for recent reviews see also Rigoni et al., 2010; Iofrida et al., 2014). Among the most commonly studied genetic polymorphisms that have been identified to influence human reactive behavior is the promoter region of the serotonin (5-HT) transporter single nucleotide polymorphism (SNP), known as 5-HTTLPR. The 5-HTTLPR polymorphism is characterized by pairs of short (S) and long (L) alleles (i.e., short/short, long/short, long/long; Lesch et al., 1996), with the short allele associated with approximately three times lower basal activity compared with the long allele (Lesch et al., 1996; Hariri et al., 2002).

Neuorimaging evidence further suggests reduced gray matter volume in both the amygdala and the perigenual cingulate cortex in individuals homozygous for the serotonin-related Short 5-HTTLPR allele, compared with high serotonin uptake long allele carriers (Pezawas et al., 2005). Given this evidence and our current understanding of the likely mechanisms and pathways via which this genetic variation impacts brain function and structure, studies which combine sensitive measures of phenotypic behavioral expressions with 5-HTTLPR and other genetic polymorphisms may be particularly valuable for elucidating the neurobiological mechanisms which contribute to human affectivity.

There is increasing evidence from the adult literature for the existence of gene-mediated pathways for looking patterns associated with emotional stimuli and contexts. Recent eye-tracking studies have examined the putative role of serotonin availability in modulating visual scanning patterns of threatening versus positive information. In an eye-tracking investigation Beevers et al. (2010) reported that adult Short 5-HTTLPR allele homozygotes selectively fixated more to positive emotional scenes when simultaneously presented with four different emotional stimuli in 30 s trials. This finding suggests selective avoidance of negative stimuli, perhaps in an effort to regulate heightened reactivity to negative stimuli. However, this finding is not consistent with evidence from behavioral studies that have measured reaction times. Most notably, during the presentation of pairs of aversive/neutral stimuli participants homozygous for the high serotonin uptake 5-HTTLPR Long allele have been observed to exhibit vigilance in response to positive and neutral stimuli, and selective avoidance when exposed to negative stimuli (Fox et al., 2009; Pérez-Edgar et al., 2010). In line with this, in a study that employed a dot-probe task involving presentations of spiders and neutral control stimuli for 2000 ms, it was found that 5-HTTLPR Short allele carriers exhibited selective preferential looking at non-social fearful stimuli (Osinsky et al., 2008). It has been suggested that the mix of findings for 5-HTTLPR effects on behavior across these studies may be related to differences in experimental design, stimuli, and dependent measures (e.g., eye-tracking versus behavioral responding) utilized across studies (see also Murphy et al., 2013, for a recent meta-analysis).

Gibb et al. (2009) reported that, in children between 8- and 14-years old, 5-HTTLPR genotype may moderate a previously documented link between maternal depression and children’s attentional biases for sad faces when presented side-by-side with neutral faces. Specifically, children of depressed mothers who themselves carry the 5-HTTLPR low serotonin-associated short or LG allele, but not those homozygous for the LA allele, exhibited attentional avoidance of sad faces (Gibb et al., 2009). However, there is a discrepancy in this area of inquiry with some studies showing that children of depressed mothers exhibit a bias reflecting preferential looking to sad faces (Joormann and Gotlib, 2007; Kujawa et al., 2011), whereas others have found evidence of avoidance (Gibb et al., 2009). In a recent study, Owens et al. (2016) directly assessed patterns of visual scanning during passive viewing of faces in an effort to determine whether preferential looking or avoidance is present in a population of children of depressed mothers. The study found that children’s genotype contributes to the nature of their visual scanning patterns. Specifically, although children of depressed mothers spent more time fixating the sad facial expression overall, this effect was moderated by normal variation in two genes associated with the hypothalamic–pituitary–adrenal (HPA) axis (Joormann and Gotlib, 2007; Kujawa et al., 2011). In particular, children of depressed mothers carrying alleles associated with low uptake of serotonin (e.g., the 5-HTTLPR Short allele) actually spent *less* time looking the sad faces, perhaps in an effort to regulate negative affectivity.

Taken together, the findings of these studies strongly suggest that genetic mechanisms associated with serotonin uptake and regulation modulate affective reactivity, but that this is not a simple or straightforward matter of genetics directly modulating behavioral responses to phylogenetically persevered stimulus or emotional features. Instead, these findings indicate that these normal genetic variations contribute to the nature and extent of reactivity to stimulus and event features which are affectively salient to the individual based, at least in part, upon their particular experience and exposures. For example, while certain SNPs may mediate the existence or extent of emotional reactivity in response to environmental exposures or events, other SNPs and previous environmental exposures may mediate the behaviorally expressed pattern, or type, of reactivity.

Another genetic SNP, BDNF Val^66^Met, has also been observed to play a role in modulating affective responses to environmental stressors. BDNF is a secreted protein which is involved in the release of neuronal survival and growth promoting factors (e.g., Wichers et al., 2008). BDNF has also been determined to play a critical role in the regulation of neural development, neural connectivity, and neural plasticity (Martinowich et al., 2007). Most relevant to the current study, the BDNF Val^66^Met polymorphisms (Bath and Lee, 2006), have been widely investigated in relation to affective disorders and associated behavioral traits. In humans these polymorphisms produce a valine-to-methionine substitution at codon 66 (Chen et al., 2006), with the Met allele associated with increased vulnerability for affective disorders (e.g., Sarchiapone et al., 2008). There is also emerging evidence suggesting increased rumination (e.g., Beevers et al., 2009) and deficits in fear conditioning (Hajcak et al., 2009) in adult carriers of the BDNF Met allele. However, only one study has been conducted to examine the role the BDNF Val66Met polymorphisms in affective reactivity in young children (Christou et al., 2015), where children carrying the low-plasticity Met BDNF allele were found to exhibit a vigilance-avoidance pattern of viewing when viewing angry (versus neutral) faces for 2500 ms. In addition, the same study reported that young children carrying the low serotonin uptake-related Short 5-HTTLPR allele spent less time fixating the eye region of neutral faces and more time fixating the mouth region.

The current study utilizes eye-tracking technology in order to examine the putative role of the common genetic variation in the serotonin transporter-linked 5-HTTLPR and neuroplasticity-related BDNF Val66Met polymorphisms in modulating fixation durations and patterns during the processing of affective social and non-social stimuli, in children aged 4–7 years. Two main hypotheses are tested as part of this study. First, taking into account previous eye-tracking evidence indicating prolonged visual scanning of negative stimuli, as well as the previously reported vigilance-avoidance patterns of processing threatening pictures in children with separation anxiety (In-Albon et al., 2010), adults with dysphoria (Caseras et al., 2007) and depression (Kellough et al., 2008), we hypothesize that the presence of elevated rates of internalizing problems in the children in the current study will be significantly correlated with vigilance-avoidance patterns of scanning negative stimuli. In particular, we hypothesize that children whose parents reported that they have elevated anxiety and depressive symptoms will initially fixate more to the negative stimuli but later will spend less dwell time fixating the same stimuli.

For the second hypothesis, taking into account emerging evidence highlighting the role of variations in the 5-HTTLPR polymorphism (Gibb et al., 2009; Beevers et al., 2010) and BDNF Val^66^Met genotype (e.g., Hajcak et al., 2009) in modulating the processing of affective stimuli, we hypothesize that carriers of the low serotonin 5-HTTLPR Short allele will exhibit a vigilance-avoidance pattern of looking for negatively valenced stimuli, compared to the high serotonin uptake Long allele group. More specifically, we predict that carriers of the low serotonin uptake Short allele will initially fixate more on the negative stimuli but during later stages of processing will spend significantly less dwell time fixating the negative stimuli, compared to Long allele homozygotes. In a similar vein, considering that carriers of the low neuroplasticity Met BDNF Val66Met allele (e.g., Hajcak et al., 2009) have been shown to exhibit increased reactivity in response to environmental stressors we hypothesize that, compared to the high neuroplasticity Val/Val group, Met allele carriers will exhibit vigilance-avoidance patterns of scanning for aversive stimuli. The over-arching aim of this study is to further our understanding of the impact of particular neurotransmitter (Serotonin) and neural plasticity (BDNF) mechanisms on behavioral reactivity for both social and non-social aversive stimulus processing during childhood.

## Materials and Methods

### Participants

Forty-nine children from Caucasian ancestry participated in the study (24 males; 25 females; Mean age in months = 70.8, *SD* = 11.5, age range 4–7 years of age). Parents or guardians of all participants reported that the child had no history of a neurological or psychiatric disorder and that they had normal or corrected to normal vision. Exclusion criterion included if participants scored below a certain range (IQ < 75) on the British Ability Scales II, Early years (BAS-II; Elliot et al., 1996), a standardized assessment of intelligence/developmental age and abilities. No participants met this exclusion criterion (see **Table 2**). All participants had English as their first language. Informed written consent was obtained from the parents/guardians of all participants before participating in the study. In addition, children aged 7 provided written assent to participate in the study. Families who expressed interest in the study were scheduled to attend a laboratory intake appointment. Families were provided with compensation of £10 toward their travel expenses. Ethical consent was gained from the University of Birmingham Ethics Committee.

### Data Collection Procedures

Children were told that they were going to see a range of interesting photos on a computer screen, while a special camera recorded their eye movements. The eye-tracking and parent report assessments took place during one laboratory visit.

#### Behavioral Measures

For the assessment of children’s behavioral problems the CBCL scales were used (Achenbach and Rescorla, 2001). Both the Early Years (for children between 1½ and 5 years of age) and School Age (children and adolescents aged 6–18 years) versions were used here. For the assessment of autism symptomatology the Social Communication Questionnaire-Lifetime Edition was completed by parents (SCQ; Rutter et al., 2003).

#### Eye-Tracking Assessment

##### Stimuli

Developmentally appropriate colored pictures that have been previously used in studies with children of the same age (Dennis and Hajcak, 2009; Solomon et al., 2011; Leventon et al., 2014) were selected from the International Affective Picture System (IAPS; Lang et al., 2008). Many studies in adults have examined preferential looking gaze patterns for emotional pictures from the IAPS picture set, which is documented to be a well standardized set of emotional stimuli. However, in studies with young children, where it was impossible to obtain subjective valence and arousal ratings for the IAPS pictures because of children’s difficulty in understanding the self-assessment mannequin rating techniques (Lang et al., 2008), a subset of developmentally appropriate IAPS pictures has been used (Dennis and Hajcak, 2009; Solomon et al., 2011; Leventon et al., 2014).

It has been previously reported that children respond in a similar manner as adults to complex developmentally appropriate subset of images/emotional stimuli from the IAPS (Lang et al., 2008). To this end, in the present study it was deemed appropriate to use a subset of IAPS pictures that had been previously used on these studies with children and adolescents, that included pleasant scenes^1^ (e.g., smiling faces, sport scenes, pleasant animals and scenes and family moments), unpleasant scenes^2^ (e.g., faces with negative expressions, attack pictures or disasters), and also neutral scenes^3^ (e.g., neutral faces, household objects or nature). Additional neutral stimuli were selected to match the requirements of the experimental design.

As in previous studies that used the same stimuli with young children, means and standard deviations for valence and arousal ratings for each picture were taken from the IAPS normative adult ratings (Lang et al., 2008).

The IAPS normative ratings are rated on a 9-point scale, where higher ratings for valence represent increased pleasantness, and higher arousal ratings correspond to more arousing stimuli. Negative, positive, and neutral pictures differed in terms of valence [Positive: Mean (*SD*) = 7.62 (1.48); Neutral: Mean (*SD*) = 5.71 (1.36); Negative Mean (*SD*) = 3.65 (1.88)]. In a similar vein, the categories of the emotional pictures differed from neutral in terms of arousal [Positive Mean (*SD*) = 4.67 (2.35); Neutral: Mean (*SD*) = 3.32 (2.07); Negative: Mean (*SD*) = 6.14 (2.01)]. A repeated measures ANOVA comparing the image categories (positive versus negative versus neutral) on valence and arousal ratings yielded a significant effect of image category on both valence ratings [*F*(2,22) = 100.28, $\eta_{p}^{2}$= 0.97, *p* < 0.001], as well as on arousal ratings [*F*(2,22) = 39.61, $\eta_{p}^{2}$= 0.94, *p* < 0.001]. As given by the means and standard deviations above, pictures with positive components had higher valence than neutral, where neutral had higher valence rates compared to negative stimuli. In contrast, negative images had higher arousal ratings than neutral, and in turn, neutral had higher arousal.

Forty-eight pairs of pictures of negative-neutral and positive-neutral pairs were selected for the present study. Stimuli pairs were approximately matched based on color, contrast, and complexity after having been reviewed by two independent investigators and were presented simultaneously side-by-side. As a general criterion, all the selected pictures can be seen by young children on a daytime television program or in the news. To investigate the potential role of social versus non-social components of the stimuli in affectivity, half of the pictures (i.e., *n* = 6) for negative and positive emotional presented scenes that involved people whereas the other half presented scenes that involved animals. In addition, the emotional-neutral pairs were matched in terms of arousal levels. The four different types of emotional pictures (i.e., negative social; negative non-social; positive social; and positive non-social) were pseudo-randomly distributed across the experiment, and each type presented equally over the left and right side of the screen. Moreover, to investigate the role of novelty in fixation durations, the first 24 pairs of the experiment consisted of novel affective stimuli (12 negative-neutral pairs, 12 positive-neutral), whereas in the second block the same emotional stimuli were each paired with a novel neutral picture. This was done to inform whether the effects of preferential looking of particular type of emotion would be able to hold across the two blocks, compared to the other types of stimuli, even when the stimuli had been previously seen.

**FIGURE 1:**
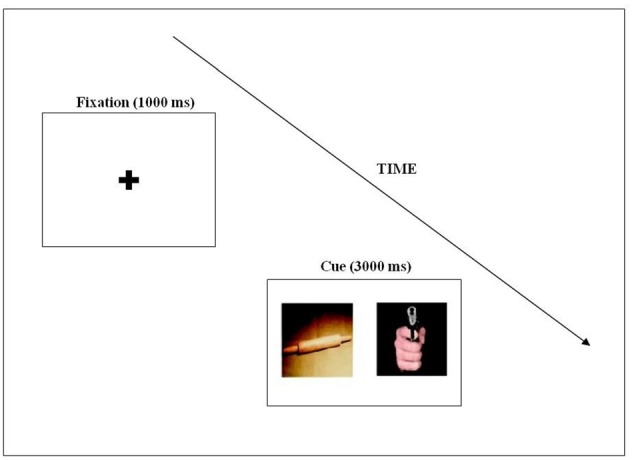
An example of the stimulus pairs used in the eye-tracking experiment, and an illustration of the trial structure.

The eye-tracking experiment was programmed using Experiment Builder software for EyeLink (SR Research, Kanata, ON, Canada). Each trial began with a fixation cross, shown for 1500 ms, measuring 2.81 × 2.08 degrees of visual angle in the middle of the screen, which was displayed for 1000 ms (except in the case of mini calibration). This was followed by a pair of pictures presented side by side for 3000 ms (see **Figure 1**). Stimuli presented on a 19-inch CRT, in 1280 × 1024 dimensions with a gap of 4.3 cm between the two pictures. Each stimulus pair was presented with a visual angle of 14.3 × 18.6 degrees.

##### Procedure

After taking consent from parents, children were escorted in a dimly lit room. Children were told that they were going to watch a range of interesting pictures on a computer screen and that if they remain still they will get a gift at the end. The assessments took place during a single laboratory visit. Participants were seated in a dimly lit room, 60 cm away from the display screen and they had their head positioned against a headrest and their chin placed on a chinrest to minimize the possibility of movements. Children were instructed to look at the pictures displayed freely. Viewing was binocular, but only data from the right eye were collected. All children initially participated on a short face processing eye-tracking experiment with an overall duration of 3-min (see Christou et al., 2015). After the completion of the first experiment, children were given a short break and were prepared to participate in the affective processing experiment. Both experiments took place during a single visit in the same experimental room using the same eye-tracking system. Children were told they were going to see different pictures on a computer screen, where their eye movements would be recorded with a special camera. During calibration, the EyeLink recorded the eye position and a 9-target location calibration was conducted providing the required reference data for computing gaze positions to ensure a point of fixation error rate of less than 0.5 degrees. A mini calibration was repeated every five trials in order to ensure that eye movement data were adjusted for small-scale movement of the head (see also Christou et al., 2015). In the case of unsatisfactory eye-tracking, a 9-point calibration was repeated. Participants then completed the experimental trials.

### Analysis

#### Analysis of Behavioral Data

For the measures of cognitive abilities (BAS-II), mean standardized IQ-scores were assessed. All children in this study had CBCL *t*-scores of less than 60 (below subclinical threshold). Raw scores from the two CBCL clusters of behavioral problems (i.e., internalizing and externalizing problems) were used for statistical analysis following the authors’ guidelines (Achenbach and Rescorla, 2001, p. 89). Autism symptomatology (SCQ) mean sum scores were calculated on the basis of raw scores. All children had an SCQ mean sum score of 12 or less.

The CBCL 1½–5 and 6–18 versions provides raw values that can be converted to age-adjusted *t*-scores if needed. All children in this study had *t*-scores of less than 60. Raw scores from the two clusters of behavioral problems (i.e., internalizing and externalizing problems) were used for statistical analysis following the authors’ guidelines (Achenbach and Rescorla, 2001, p. 89). Higher total scores in each CBCL subscale suggest the existence of more problematic behaviors. Autism symptomatology (SCQ) mean sum scores were calculated on the basis of raw scores.

#### Reduction of Eye-Tracking Data

Data analysis aimed to measure the time course of preferential looking toward and away positive and negative emotional information, relative to the neutral stimulus.

Fixations were calculated using the EyeLink online detection analysis algorithm when eye movement met the following four criteria: (a) velocity threshold of 30°/s, (b) a motion threshold of 0.1°, (c) a 8000°/s^2^ acceleration threshold, (d) and the pupil was not missing consecutively for three or more times from a sample. Trials were classified as ‘invalid’ if a child did not look at all at the stimuli during the trial. In addition, if more than 40% invalid trials were evident the participant’s data were excluded from further analyses [Mean invalid trials (*SD*) = 1.4 (1.3)]. No participant met this exclusion criterion; therefore, all 49 participants provided valid eye-tracking data.

For the investigation of the fixation durations toward and away positive and negative emotions, dwell time was calculated in ms after subtracting the overall dwell time of the neutral stimuli from the emotional stimuli. The eye-tracker (EyeLink 1000, SR Research) sampled eye position at 500 Hz (i.e., every 2 ms). Average spatial accuracy is between 0.25° and 0.5° of visual angle. This was done separately for each subject and for each positivity and negativity-inducing trial. Average dwell time of looking for each emotion type (i.e., negative and positive) was later calculated for each subject. A separate calculation of social versus non-social trials, for the first and second block separately, was also conducted for each emotion type. After the subtraction, the positive values represented preference toward the emotion and negative values avoidance of the emotional stimuli.

Aiming to remain consistent with previous studies measuring proportion of fixations to emotional stimuli in adults (Rohner, 2002) and in children (Gamble and Rapee, 2009) where the 3-s stimulus exposure was divided into 1-s, in the present study proportion of fixations to the emotional picture was computed for each emotion type and each 1000 ms time interval. In line with this notion, recent meta-analytical reviews in the field of affective processing suggesting that the vigilance in the content of vigilance-avoidance hypothesis has been particularly captured after 500 ms of presentation when multiple stimuli compete for attention (for a review see Weierich et al., 2008), which has been suggested to be due to initial fixation on a stimulus within the 0–1000 ms epoch (for a recent review see Armstrong and Olatunji, 2012). Together, taking into account the above evidence and due to the complexity of the affective stimuli and developmental age of the participant, the selection of three equal 1 s time windows for the investigation of the vigilance-avoidance patterns of scanning affective stimuli was deemed as the most appropriate in the present study.

#### Analysis of Genetic Material

##### 5-HTTLPR Genotyping

Direct bidirectional Sanger sequencing was used to genotype the 5-HTTLPR polymorphism. The region containing the 43 bp insertion polymorphism was amplified using primers described (Hu et al., 2006) producing a 528 bp amplification product from the L allele and a 485 bp product from the S allele. Polymerase Chain Reaction (PCR) was performed using Megamix PCR solution (supplied by Microzone UK Ltd.) in a total volume of 25 μl, containing 25 pmol of each primer and 3 μl of betaine. An initial denaturation step at 95°C for 5 min was followed by 30 cycles of PCR (95°C 1 min, 58°C 1 min, and 72°C 1 min) and then a final extension at 72°C for 10 min. PCR products were purified using Exonuclease I and Shrimp Alkaline Phosphatase (according to manufacturer’s instructions). Ten microliter sequencing reactions were generated containing 0.25 μl BigDye Terminator (v3.1, Applied Biosystems), 1.9 μl molecular grade water, 3 pmol of forward or reverse primer, and 1 μl purified HTTLPR PCR amplicon (diluted 1 in 2). Cycle conditions for sequencing included an initial denaturation step at 95°C for 5 min followed by 30 cycles of (95°C 30 s, 50°C 10 s, and 60°C 4 min) and reaction products were purified using CleanSEQ beads (Agencourt) in a 1:1 ratio as described by the manufacturer. Products were re-suspended in 70 μl molecular grade water and analyzed on a 3730 Genetic Analyser (Applied Biosystems).

Allele frequencies across participants for 5-HTTLPR was *n* = 42 (42.8%) for Short allele and *n* = 56 (57.2%) for Long Allele. To this end three genotype groups where resulted, one with Short allele homozygotes (i.e., S/S; *N* = 10), heterozygotes L/S (*N* = 22), as well as homozygotes for the Long allele (i.e., L/L; *N* = 17). 5-HTTLPR genotype frequencies where in Hardy-Weinberg equilibrium [*x*^2^(1) = 0.340, *p* = 0.559] as calculated using an online tool: http://www.oege.org/software/hwe-mr-calc.shtml.

Carriers of at least one Short allele [i.e., Heterozygotes (S/L), and Homozygotes for the Short allele (S/S)] (Court, Court), were grouped in one ‘Short allele carriers’ group (i.e., S/-; *N* = 32) and compared with the remaining homozygous participants for the high serotonin uptake Long allele (L/L; *N* = 17). Additional classifications with two (i.e., S/S versus L/-) and three genotype groups where also employed (i.e., L/L versus L/S versus S/S).

##### BDNF Genotyping

Direct bidirectional Sanger sequencing was used to genotype the SNP within the BDNF gene (rs6265). PCR primers were designed to flank the polymorphism producing a 249 bp amplification product. Sequences of the primers are as follows: forward AAACATCCGAGGACAAGGTG and reverse AGAAGAGGAGGCTCCAAAGG. PCR was performed using Megamix PCR solution (supplied by Microzone UK Ltd.) in a total volume of 25 μl containing 25 pmol of each primer. An initial denaturation step at 95°C for 5 min was followed by 30 cycles of PCR (95°C 1 min, 58°C 1 min, and 72°C 1 min) and then a final extension at 72°C for 10 min. PCR products were purified and sequenced as described for the 5-HTTLPR genotyping.

Allele frequencies for the BDNF Val^66^Met was *n* = 24 (25%) for Mel alleles and *n* = 74 (75 %) for Val alleles, respectively. To this end, three genotype groups resulted; one with Met allele homozygotes (i.e., M/M; *N* = 3), heterozygotes (i.e., V/M; *N* = 18), as well as homozygotes for the Val allele (i.e., V/V; *N* = 28). BDNF Val^66^Met genotype frequencies where in Hardy-Weinberg equilibrium [*x*^2^(1) = 0.002, *p* = 0.962] as calculated using an online tool: http://www.oege.org/software/hwe-mr-calc.shtml.

Similarly to the practice for the 5-HTTLPR genotype groupings, carriers of at least one Met allele [i.e., Heterozygotes (Met/Val), and Homozygotes for the Met allele (Met/Met)], were grouped in one ‘Met allele carriers’ group (i.e., M/-). Additional classifications with three genotype groups where also employed (i.e., V/V versus V/M versus M/M).

#### Statistical Analysis

##### Preliminary Analyses

Descriptive statistics were conducted in order to describe the sample’s demographic characteristics such as, gender, age, and distribution of cognitive abilities. Raw data from the behavioral and cognitive scales were examined for normality using Kolmogorov–Smirnov tests. The CBCL subscales did not met criteria for normal distributions (Kolmogorov–Smirnov, *p* < 0.005). Therefore, to further examine possible correlations between age, gender, IQ, and scores on the behavioral measures, Spearman’s Rho non-parametric correlations coefficients tests were performed. Moreover, Pearson correlation analyses were conducted to determine if a correlation among demographic characteristics or cognitive performance and genotype group was evident, and Spearman correlation analyses were conducted to investigate possible correlations between 5-HTTLPR and BDNF Val^66^Met Genotypes and demographic, cognitive, and rates of affective problems in the sample.

##### Behavioral Ratings and Eye Gaze Patterns

The primary analysis examined whether children’s behavioral scores (i.e., early behavioral problems; rates of autism symptomatology) were correlated with visual scanning patterns in response to particular emotional picture. Therefore, initial parametric and non-parametric correlation analyses were conducted with the overall viewing dwell time to the negatively and positively valenced pictures separately.

##### Genetics and Visual Scanning

Each 3-s trial was divided into three equal 1000 ms intervals. The relative viewing dwell time (in ms) of the emotional images was calculated for each 1000 ms interval and then averaged across trials for each emotion (i.e., positive versus negative), condition (i.e., social versus non-social), and block (i.e., block 1 versus block 2) separately. A 2 (Image Type: negative versus positive) by 3 (Time: 0–1000 ms versus 1001–2000 ms versus 2001–3000 ms) by 2 (Condition: social versus non-social) by 2 (Block: novel emotional versus familiar emotional) mixed analysis of variance (ANOVA) with 5-HTTLPR Genotype (L/L versus S/) and Gender as between-groups factors was conducted. The same analysis was repeated separately with different 5-HTTLPR genotype classification (i.e., S/S versus S/L versus L/L), as well as with two (i.e., V/V versus M/-) and three (M/M versus V/M versus V/V) BDNF Val^66^Met genotype. All within subject, effects that violated the assumption of sphericity were adjusted using the Greenhouse-Geisser correction (adjusted degrees of freedom are noted as adj. df). To further evaluate the time course of attention allocation, independent samples *t*-tests were conducted to determine whether there was a looking preference toward or away from the emotional images of a specific genotype group at any of the 1000 ms time intervals. This was done for each SNP (i.e., 5-HTTLPR and BDNF Val^66^Met) and each emotion, separately, after the initial ANOVA. When the data did not satisfy Kolmogorov–Smirnov tests for normality, Mann-Whitney *U* tests were performed instead of *t*-tests.

## Results

### Demographic Characteristics

**Tables 1, 2** present the participants’ main demographic characteristics, including gender, age, and cognitive ability. Correlation analyses did not reveal any significant correlation between demographic characteristics and behavioral measures, or correlations between demographics, rates of early behavioral problems and genotype.

**Table 1:** Sample size and demographic characteristics of sample.

*N*		49
Gender	% Male *(N)* % Female *(N)*	48.9 *(24)* 51.1 *(25)*
Handedness	% Right *(N)* % Left *(N)*	77.3*(39)* 22.7*(10)*
SCQ Total Score	Mean *(SD)* Range	3.63*(2.77)* 0–12
BAS-II Total Score	%Below average % Average %Above average % High	3.8 65.4 25.0 5.8

**Table 2:** Participants’ general and age-equivalent cognitive abilities.

	Mean *(SD)*	Range
Chronological Age^∗^	70.8 *(11.5)*	55–91
Overall Ability^∗∗^	106.8 *(8.7)*	86–125
Verbal Ability	103.5 *(13.9)*	58–127
Non-verbal Ability	110.8 *(13.8)*	86–144
Developmental Age^∗^	63.9 *(13.1)*	42–88
Developmental Verbal Ability (Months)	64.9 *(15.5)*	35–96
Developmental Non-Verbal Ability (Months)	66.6 *(15.5)*	35–96

*^∗^Age data presented in months.*

*^∗∗^Overall ability is calculated from the overall BAS-II total score and Verbal and Non-verbal ability form the BAS-II clusters of abilities. Values represent GCA.*

Moreover, *t*-tests showed that the two 5-HTTLPR genotype groups did not differ in terms of Age [*t*(47) = -0.037, *p* = 0.971], Gender [*t*(47) = 0.994, *p* = 0.325], IQ [*t*(47) = -1.17, *p* = 0.245], developmental age [*t*(47) = -0.245, *p* = 0.808], or other behavioral measures. Similarly, the two BDNF Val^66^Met genotype groups did not differ in terms of Age [*t*(47) = 0.000, *p* = 1.00], Gender [*t*(47) = 0.162, *p* = 0.872], IQ [*t*(47) = -0.427, *p* = 0.671], or developmental age [*t*(47) = -0.223, *p* = 0.824].

Task engagement was calculated by subtracting the relative looking time away from the areas of the stimuli from the time looking the affective stimuli. More specifically, the dwell time corresponding on the time spent on the each affective stimuli, was subtracted from the time spent looking other than the affective areas on the screen for each trial. After the subtraction, the positive values represented preference toward the emotion and negative values avoidance of the emotional stimuli. This analysis show that participants spent consistently over 60% of the time looking the affective stimuli [Mean (*SD*) = 0.625 (0.31)] and a *T*-test test show that these rates did not differ between the 5-HTTLPR S/- and L/L genotypes [*t*(47) = 0.436, *p* = 0.665] as well as when comparing three 5-HTTLPR genotypes (one-way ANOVA; *p* = 0.320). In a similar vein no difference on the task engagement rate where evident between the two [*t*(47) = 0.156, *p* = 0.887] or three BDNF genotypes (one-way ANOVA; *p* = 0.721). Moreover, repetition of the same analysis for the engagement with negative and positive stimuli separately showed these rates did not differ between the 5-HTTLPR S/- and L/L genotypes [*t*(47) = 0.432, *p* = 0.661] as well as when comparing three 5-HTTLPR genotypes (one-way ANOVA; *p* = 0.622). In a similar vein no difference on the task engagement rate where evident between the two [*t*(47) = 0.425, *p* = 0.772] or three BDNF genotypes (one-way ANOVA; *p* = 0.522).

### Behavioral Effects in Fixation Duration

Pearson correlation analyses revealed a negative correlation between looking time of the negatively valenced stimuli and age (*r* = -0.559, *p* < 0.001), where younger children exhibited higher reactivity/attenuation toward negative stimuli. No further significant correlations between children’s demographic characteristics and fixation duration for each emotion, block, condition, and time point were observed. Moreover, no significant correlation was evident with children’s internalizing and externalizing problems and average fixation duration at any emotion, time point, condition or block (See Appendix 7).

### Genotype Effects in Fixation Duration toward Affective Stimuli

A 2 (Image Type: negative versus positive) by 3 (Time: 0–1000 ms versus 1001–2000 ms versus 2001–3000 ms) by 2 (Condition: social versus non-social) by 2 (Block: novel emotional versus familiar emotional) mixed ANOVA with Genotype (5-HTTLPR L/L versus S/-) and Gender as between factors revealed a significant main effect on Emotion [*F*(1,45) = 6.27, $\eta_{p}^{2}$= 0.122, *p* = 0.016], where participants exhibited a preferential looking pattern toward the positive stimuli compared to the negatively stimuli (see **Table 3**; see also Appendix 1 for raw data). A significant main effect of Time was also evident [*F*(2,44) = 121.80, $\eta_{p}^{2}$= 0.730, *p* < 0.001] with visual scanning duration to be evident to peak at T_2_ [1001–2000 ms] and declined on the later stage of processing (see Appendix 2). Moreover, a main effect of Block [*F*(1,45) = 112.72, $\eta_{p}^{2}$= 0.715, *p* < 0.001] suggests that participants during Block 2 spent less time looking on the emotional/previously seen emotional stimuli (Block 1) and compensate the time by exploring the novel neutral stimuli (see **Table 3**). In addition, a significant two-way Time by Block interaction [*F*(2,44) = 44.66, $\eta_{p}^{2}$= 0.498, *p* < 0.001] suggests that independently of the emotion valance children spent less time looking the emotional/previously seen stimuli on the second block, and spend more time exploring the novel neutral stimuli (see Appendix 2). Paired samples *t*-test have been conducted to further investigate on which time point this pattern was more pronounced showing significant reductions on the time spent looking the affective stimuli at T1 [*t*(48) = 3.86, *p* < 0.001], T2 [*t*(48) = 12.30, *p* < 0.001], and T3 [*t*(48) = 7.44, *p* < 0.001]. However, since factors such as fatigue or excitement of young participants may have induced non-compliance-effects at the beginning, and the absence of full randomisation of novelty across the experiment, the present effects would be necessary to be interpreted with caution.

**Table 3:** Participants’ mean time (in ms) and standard deviations (in brackets) spent per emotion, condition, and block, averaged across time points.

	Social		Non-Social	
	Block 1	Block 2	Block 1	Block 2
**Positive**	495 *(278)*	200 *(298)*	588 *(325)*	*243 (299)*
**Negative**	448 *(364)*	140 *(363)*	344 *(436)*	*22 (354)*

*Dwell time was calculated for each subject and type of stimuli in ms after subtracting the overall dwell time of the neutral stimuli from the emotional stimuli.*

Furthermore, a two-way Emotion by Time interaction effect was evident [*F*(2,44) = 6.72, $\eta_{p}^{2}$= 0.130, *p* = 0.002] where participants spent more time looking at the positive stimuli across blocks and conditions relatively to the negative stimuli, difference which was more pronounced over T_2_ (1001–2000 ms; see Appendix 2). Similarly, a two-way Emotion by Condition effect [*F*(1,45) = 6.03, $\eta_{p}^{2}$= 0.118, *p* = 0.018] was observed with relatively lower visual scanning time to be evident for non-social negative stimuli (see **Table 3**). In addition, a three-way Emotion by Time by Condition interaction was observed [*F*(2,44) = 5.69, $\eta_{p}^{2}$= 0.112, *p* = 0.005] with more time spent looking at the positive non-social stimuli (i.e., happy animals; sweets) than social and/or negative, which was more pronounced during 2001–3000 ms (T_3_; see Appendix 2). A repetition of the same ANOVA analysis as above, including age as a between factor, showed an additional effect of Emotion by Time by Age [*F*(1,50) = 3.26, $\eta_{p}^{2}$= 0.118, *p* = 0.006], where younger children exhibited higher reactivity/attenuation toward negative stimuli. No additional effects of age were evident, while the originally reported effects have remained intact.

**FIGURE 2:**
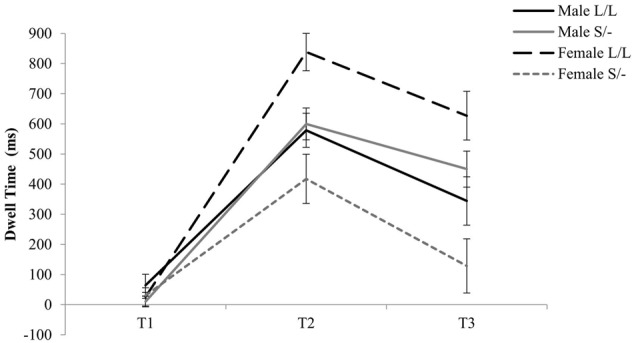
Genotype and gender effects on time course of visual scanning of emotional stimuli.

With regards to the effects of serotonin transporter polymorphism, a two-way Time by 5-HTTLPR genotype was evident [*F*(2,90) = 3.61, $\eta_{p}^{2}$= 0.074, *p* = 0.031] where Short allele carriers, compared to Long allele homozygotes, spent less time looking at the emotional stimuli independently of valence during T_2_ (1001–2000 ms; see **Figure 2**). Moreover, a three-way Time by 5-HTTLPR by Gender interaction was evident [*F*(2,90) = 10.79, $\eta_{p}^{2}$= 0.193, *p* < 0.001] where homozygous female participants for the high serotonin uptake Long allele spent significantly more time processing the emotional stimuli independently of the valence at T_2_ (Mean = 838.53; *SD* = 163.10; see **Figure 2**) and female Short allele carriers spent less dwell time looking at the emotional stimuli during T_2_ (Mean = 417.61, *SD* = 345.77). No further effects of the 5-HTTLPR were evident for this analysis.

To further investigate the two-way Time by 5-HTTLPR genotype (S/- versus L/L) interaction effect, the dwell time at each of the three time points was averaged across the two emotions. A Kolmogorov–Smirnov test of normality showed that the averaged data at each time point was normally distributed (*p* > 0.005). Therefore, a *t*-test was conducted at each time point, which showed Short allele carriers spent significantly less time looking at the emotional stimuli when compared to Long allele homozygotes during 1001–2000 ms [*t*(47) = -2.28, *p* = 0.027; see Appendixes 4–6]. Interestingly, a two-way ANOVA at each of the three time points of processing emotional stimuli irrespectively of the valance, with both gender and 5-HTTLPR genotype as independent variables, show a significant Gender by 5-HTTLPR effect at T2 [*F*(1) = 7.94, *p* = 0.007, $\eta_{p}^{2}$= 0.150] and T3 [*F*(1) = 11.15, *p* = 0.002, $\eta_{p}^{2}$= 0.199]. This may suggest that gender when combined with genetic mechanisms may modulate behavioral reactivity in response to emotional information early in life.

**FIGURE 3:**
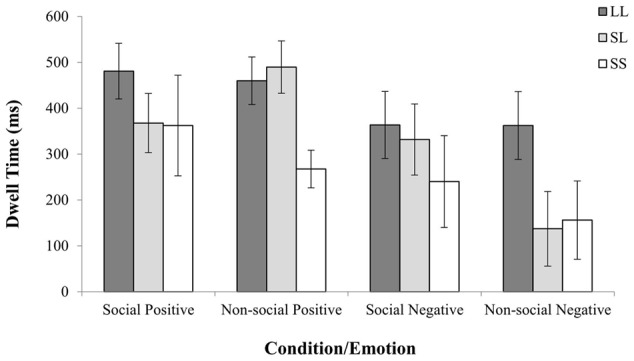
5-HTTLPR genotype effects on relative viewing time per emotion and condition. The presence of one Short allele was associated with avoidance pattern of non-social negative stimuli, whereas two copies of the genotype with two copies of the Short allele were associated with reduced looking at non-social positive stimuli.

This initial analysis was repeated with three 5-HTTLPR genotype groups (i.e., L/L versus L/S versus S/S) which revealed an additional three-way Emotion by Condition by 5-HTTLPR interaction [*F*(2,43) = 1.78, $\eta_{p}^{2}$= 0.159, *p* = 0.024]. This analysis suggested that participants who were carriers of two copies of the Long allele, spent more time exploring the non-social threatening stimuli, where Short allele carriers (i.e., S/S and L/S) spent relatively less time looking at the negatively valenced non-social stimuli (see **Figure 3** and Appendix 2).

The initial ANOVA was repeated with the BDNF Val^66^Met genotype (i.e., V/V versus M/-), which did not revealed any Time × BDNF genotype effect [*F*(2,90) = 0.30, $\eta_{p}^{2}$= 0.030, *p* = 0.506] or any additional effect (see Appendixes 4–6). Similarly, when entering the three BDNF genotype groups (i.e., M/M versus V/M versus V/V) as between factor, no significant Time by BDNF interaction [*F*(4,86) = 0.55, $\eta_{p}^{2}$= 0.025, *p* = 0.693] or a three-way Emotion by Condition by BDNF Genotype [*F*(2,43) = 1.10, $\eta_{p}^{2}$= 0.005, *p* = 0.896], or any other interaction was evident (see also Appendix 3).

To further delineate the 5-HTTLPR genotype effects on fixation duration toward social and non-social fearful stimuli, separate one-way ANOVAs were conducted after the relative dwell time was averaged for Negative Non-Social, Positive Social, and Positive Non-Social stimuli (normally distributed; Kolmogorov–Smirnov test *p* > 0.05) comparing the three 5-HTTLPR genotype groups. Since the data from the average fixation duration in response to Negative Social stimuli were not normally distributed (Kolmogorov–Smirnov test *p* < 0.05), a Kruskal–Wallis test was conducted. The ANOVAs revealed a significant effect of 5-HTTLPR genotype on the time spent looking the negative non-social stimuli [*F*(2) = 4.04, *p* = 0.025] showing that Long allele homozygotes spent significantly more time looking at the non-social negative stimuli (see **Figure 3** and Appendix 2). In contrast, the ANOVAs did not revealed any significant effect for the non-social positive stimuli [*F*(2) = 2.66, *p* = 0.080] and social positive stimuli [*F*(2) = 0.844, *p* = 0.437]. In a similar vein the Kruskal–Wallis test did not revealed any significant effect of 5-HTTLPR genotype on the processing of negative stimuli with social component [*x*^2^(2) = 2.21, *p* = 0.330].

Interestingly, however, a separate Mann Whitney-U analysis with a different classification of genotype (S/S vs L/-) revealed an effect of genotype on scanning non-social stimuli with positive valence (U = 0.94, p = 0.011) (see **Figure 3**). Taken together, the above findings suggest that currying two copies of the Long allele (L/L) was consistently associated with more time spend scanning both positively and negatively valenced non-social stimuli, while carriers of the two copies of the Short allele (S/S) consistently spent less time fixating the non-social stimuli.

## Discussion

The aim of the current study was to examine the impact of normal variation in genetic SNPs known to impact serotonin availability and neural plasticity mechanisms on social and non-social affective stimulus processing. First, the study reported lower visual scanning allocation in response to negative non-social stimuli (e.g., animals) during the later stage of visual scanning. This pattern is consistent with a plethora of neurophysiological studies of adults that have highlighted the reduced role of non-social fear in generating affective neural responses (e.g., Prather et al., 2001; Kirsch et al., 2005), and the current findings extend this into children.

With regards to effects of genotypes, the study findings indicate that children with two copies of the Short 5-HTTLPR allele exhibited a consistent pattern of spending less time fixating non-social stimuli with either a positive or negative affective component. On the other hand, carriers of two copies of the Long allele spent more time fixating both positive and negative non-social stimuli compared to participants with two copies of the short 5-HTTLPR allele. However, follow-up analysis with three 5-HTTLPR genotype groups indicated that only the L/L genotype was significantly associated with more time viewing the non-social negative stimuli, compared to S allele carrier groups. This is partially consistent with the study hypothesis. Our study hypothesis was that carriers of the low serotonin uptake Short allele would initially fixate more toward the negative stimuli but then, during later stages of processing, spend significantly less dwell time fixating the negative stimuli, compared to Long allele homozygotes. The current findings support an avoidance association between 5-HTTLPR genotype and affective stimulus processing for both positively and negatively valenced stimuli. Together, the findings are consistent with the literature in which 5-HTTLPR genotype has been reported to differentially modulate amygdala responses for both negative and positive emotional stimuli (e.g., Dannlowski et al., 2010).

In a recent study, we examined the impacts of 5-HTTLPR genotype on attention patterns for face stimuli in the same group of children (Christou et al., 2015). In this study, we did not observe reduced scanning of negative or positive emotional faces in carriers of the Short allele. However, we did observe reduced looking to the eye region in carriers of the Short allele. Eyes are one of the most salient features of the face, provide critical information about facial emotions (Gold et al., 2008), and the eyes serve as a critical “base” for foveal focus during face processing (e.g., Baron-Cohen et al., 1997). Therefore, one possibility is that a characteristic of 5-HTTLPR Short allele carriers is to be more reactive to the most salient and informative environmental stimuli. Thus, in the absence of a single critical stimulus feature (e.g., eyes) in the current stimulus set, 5-HTTLPR Short carriers were reactive to the negative and positive, versus neutral, affective stimuli. Regardless, the findings from the current and previous study are consistent in that the 5-HTTLPR Short carriers exhibit reactive avoidant looking patterns for salient stimuli across the two studies. Further research is required in order to delineate the neural and behavioral mechanisms underlying differential reactivity patterns for emotional faces and scenes.

In addition to the effect of 5-HTTLPR genotype in modulating visual scanning pathways in response to non-social affective stimuli, an unanticipated interaction between serotonin genotype and gender was evident in the time course of viewing emotional stimuli, irrespectively of the valence. More specifically, compared to females carrying the Short allele and males with either genotype, females with two copies of the Long 5-HTTLPR allele spent significantly more time looking at the emotional stimuli during T_2_ and T_3_. The current observation for gender by genotype interaction suggest the possibility of a gender-specific biological contribution to the visual scanning of affective stimuli early in life, which in turn may also relate to increased susceptibility for behavioral problems. Although research has previously highlighted the existence of gender differences in the manifestation of affectivity, especially in relation to depression, where increased susceptibility for affective disorders has been found in females compared to males (Nestler et al., 2002), the particular gender underpinnings that may influence susceptibility for affective disorders are currently unknown (e.g., for a review see Bale, 2006). Furthermore, given that there is no consistent or conclusive evidence for gender effects on affectivity as measured by eye-tracking, as well as the limited sample size for gender comparisons in the current study, the present pattern of findings should be interpreted with caution. On the other hand, the current findings do suggest that eye-tracking may be sensitive to gender differences in emotional reactivity early in life, which warrants further investigation.

As part of the present study, and based in part upon our previous results (Christou et al., 2015), it was hypothesized that, compared to the high neuroplasticity Val/Val group, Met allele carriers would exhibit vigilance-avoidance patterns of scanning for the aversive stimuli. However, in contrast to the study’s hypothesis, the results did not show any significant impact of the BDNF Val^66^Met genotype on the visual scanning of affective stimuli (e.g., Montag et al., 2008; Schofield et al., 2009; Lau et al., 2010). As indicated earlier, there is a possibility that differing biological and genetic mechanisms may drive reactivity in response to faces versus aversive scenes. Furthermore, these mechanisms may be undergoing significant maturation during these sensitive periods. For example, it is possible that emotional face processing involves specific neurobiological mechanisms with a unique developmental time course which may differ from affective scene processing (for a review see Nelson, 2001).

An interesting complementary or alternative explanation of the present pattern of findings is the existence of reward-seeking behavior in carriers of the high serotonin uptake-related Long 5-HTTLPR allele. More specifically, the pattern of the eye movement behavior suggest that carriers of two copies of the Long allele consistently spent more time fixating the different types of negative but also positive stimuli, compared to carriers of the Short allele. This pattern of findings reaches statistical significance during the processing of non-social stimuli. Considering previous studies which have reported positive associations between the presence of the Long 5-HTTLPR allele and novelty seeking behaviors (e.g., Strobel et al., 2003) future research involving specific manipulations of reward and novelty seeking across 5-HTTLPR genotypes will be critical for differentiating between neuropsychological indexes of vigilance-avoidance and reward and novelty seeking, as well as positive approach.

An additional hypothesis of the current study was that children whose parents report that they have elevated anxiety and depressive symptoms will initially fixate more to the negative stimuli but later will spend less dwell time fixating the same stimuli. We did not observe significant correlations between parent reports of early affective problems, including anxiety traits, and average fixation duration to emotional pictures. Therefore, previously documented association between early affective traits and atypicalities on visual scanning behaviors in youths are not confirmed here (Martin et al., 1992; Vasey et al., 1995, 1996; LoBue and Pérez-Edgar, 2014). As highlighted earlier, the sample of children that was employed in the present study consists of a young unaffected population. Therefore, the regulatory mechanism of visual scanning may differ between healthy children, as in the study’s sample, and affected subgroups (e.g., LoBue and Pérez-Edgar, 2014). However, the fact that children in each genotype group were matched on their early affectivity rates, allows for the outcomes coming from the eye movement data to be uniquely attributed to the variations of the serotonin transporter-linked 5-HTTLPR polymorphism.

In summary, the current study provides evidence to suggest that carriers of at least one Short 5-HTTLPR allele exhibited increased reactivity in response to threat-related non-social stimuli, compared with participants with two copies of the Long allele. Moreover, a separate set of analyses highlighted that carriers with two copies of the short 5-HTTLPR allele also spent more time fixating both the negative and positive non-social stimuli. Overall, these results are consistent with existing evidence from the adult, adolescent, and child psychopathology research literatures which suggest a contribution of 5-HTTLPR genotype to variations in affective and emotional regulation. In the context of this previous research, the current findings provide critical support for the role of serotonin in mediating affective reactivity in young children. The current findings also suggest a need for more direct research on the putative roles of both social versus non-social stimulus contexts and gender in mediating the interaction between 5-HTTLPR genotype and reactive visual scanning, across development.

## Author Contributions

AC and JM developed the study concept and design. Testing and data collection were performed by AC. Genetic analyses performed by YW, HB, and MZ. Data analysis was performed by AC and JM. AC and JM wrote the manuscript. All authors approved the final version of the manuscript for submission.

## Conflict of Interest Statement

The authors declare that the research was conducted in the absence of any commercial or financial relationships that could be construed as a potential conflict of interest.

## Footnotes

1 The IAPS numbers for pleasant pictures are: 1460, 1610, 1710, 1920, 2070, 2091, 2224, 7325, 7330, 7400, 8031, and 8496.

2 The IAPS numbers for threatening pictures are: 1050, 1120, 1201, 1300, 1321, 1930, 3280, 6190, 6300, 9490, 9582, and 9594.

3 The IAPS numbers for neutral pictures are: 5130, 5210, 5220, 5201, 5250, 5390, 5551, 5611, 5631, 5711, 5740, 5750, 5800, 5820, 5870, 5890, 5900, 7002, 7000, 7004, 7009, 7010, 7025, 7030, 7031, 7035, 7041, 7050, 7100, 7140, 7150, 7175, 7190, 7224, 7233, 7235, 7236, 7496, 7512, 7502, 7545, 7560, 7580, 7595, 7632, 7705, 7900, and 7950.
