# Supplementary material for: Serotonin 5-HTTLPR Genotype Modulates Reactive Visual Scanning of Social and Non-social Affective Stimuli in Young Children
Source: Front Behav Neurosci. 2017 Jun 23;11:118. doi: 10.3389/fnbeh.2017.00118 (PMC5482294; doi:10.3389/fnbeh.2017.00118)
Supplement: Supplementary file 1 [file Data_Sheet_1.docx]

Appendix 1. Participants’ mean time (in ms) and standard deviations (in brackets) spent per emotion, condition and block, averaged across time points.

|  | **Social** | | **Non-Social** | |
| --- | --- | --- | --- | --- |
|  | **Block 1** | **Block 2** | **Block 1** | **Block 2** |
|  |  |  |  |  |
| **Positive** | 1851*(334)* | 1105 *(238)* | 1836 *(383)* | 1247*(269)* |
| **Negative** | 1416 *(231)* | 1008*(205)* | 1295 *(134)* | 575 *(227)* |

Appendix 2. 5-HTTLPR genotype groups dwell time (in ms) and standard deviations (in brackets) per Emotion, Block, Condition and Time Points. Carriers of at least one Short allele are spending less time fixating negative stimuli overall, across blocks, different which is more pronounced for the non-social threat stimuli.

|  | | **Block 1** | | | | | |  | **Block 2** | | | | | |
| --- | --- | --- | --- | --- | --- | --- | --- | --- | --- | --- | --- | --- | --- | --- |
|  |  | **Social** | | | **Non-Social** | | |  | **Social** | | | **Non-Social** | | |
|  |  | **T1** | **T2** | **T3** | **T1** | **T2** | **T3** |  | **T1** | **T2** | **T3** | **T1** | **T2** | **T3** |
|  |  |  |  |  |  |  |  |  |  |  |  |  |  |  |
| L/L  S/L  S/S | Negative  Positive  Negative  Positive  Negative    Positive | 80  *(149)* | 905  *(448)* | 719  *(586)* | 129  *(207)* | 945  *(602)* | 617  *(626)* |  | -7  *(140)* | 368  *(500)* | 117  *(514)* | 8  *(74)* | 296  *(401)* | 178  *(543)* |
|  |  | 44  *(163)*  97  *(140)*  97  *(113)* | 925  *(398)*  743  *(541)* 879  *(425)* | 680  *(403)*  387  *(680)* 468  *(391)* | 79  *(228)*  91  *(116)* 48  *(140)* | 1065  *(331)*  478  *(632)* 941  *(516)* | 877  *(516)*  69  *(806)* 769  *(606)* |  | 23  *(116)*  -50  *(87)*  -6  *(92)* | 501  *(385)*  334  *(534)* 353  *(495)* | 274  *(434)*  77  *(703)* 144  *(549)* | 35  *(110)*  -19  *(128)*  9  *(62)* | 479  *(428)*  -95  *(525)* 513  *(435)* | 225  *(499)*  -186  *(634)* 408  *(467)* |
|  |  | -18  *(112)*  -9  *(114)* | 576  *(481)*  740  *(416)* | 439  (*468)*  560  *(593)* | 39  *(183)*  -3  *(130)* | 522  *(417)*  804  *(298)* | 215  *(553)*  538  *(492)* |  | 0  *(98)*  -38  *(83)* | 336  *(538)*  258 *(405)* | 107  *(558)*  290  *(541)* | 39  *(86)*  -34  *(112)* | 156  *(415)*  218  *(311)* | -35  *(592)*  81  *(488)* |

Appendix 3. BDNF genotype groups mean dwell time (in ms) and standard deviations (in brackets) per Emotion, Block, Condition and Time Points. No significant variations between the two genotypes observed.

|  | | **Block 1** | | | | | | **Block 2** | | | | | |
| --- | --- | --- | --- | --- | --- | --- | --- | --- | --- | --- | --- | --- | --- |
|  |  | **Social** | | | **Non-Social** | | | **Social** | | | **Non-Social** | | |
|  |  | **T1** | **T2** | **T3** | **T1** | **T2** | **T3** | **T1** | **T2** | **T3** | **T1** | **T2** | **T3** |
| V/V | Negative | 83  *(134)* | 825  *(458)* | 527  *(503)* | 132  *(171)* | 669  *(597)* | 295  *(701)* | -40  *(94)* | 398  *(494)* | 149  *(492)* | 5  *(116)* | 181  *(387)* | 86  *(451)* |
|  | Positive | 111  *(111)* | 966  *(433)* | 546  *(481)* | 85  *(160)* | 938  *(427)* | 762  *(617)* | -5  *(101)* | 399  *(466)* | 198  *(525)* | 10.59  *(95)* | 472  *(330)* | 338  *(438)* |
| M/V  M/M | Negative  Positive  Negative  Positive | 59  *(168)*  6  *(134)*  15  *(63)*  -102  *(177)* | 668  *(605)*  733  *(343)*  760 *(289)*  580 *(188)* | 440  *(795)*  624  *(417)*  750  *(574)*  274  *(207)* | 55  *(161)*  17  *(194)*  -25  *(60)*  -63  *(130)* | 546  *(663)*  1007  *(426)*  804  *(363)*  828  *(567)* | 221  *(814)*  820  *(497)*  309  *(482)*  317  *(213)* | 12  *(114)*  -10  *(107)*  -114  *(198)*  64  *(41)* | 268  *(586)*  420  *(439)*  313  *(329)*  46  *(214)* | -44  *(775)*  312  *(489)*  376  *(285)*  -175  *(403)* | -3  *(85)*  26  *(78)*  910  *(172)*  -101  *(137)* | -27  *(635)*  437  *(539)*  0  *(323)*  58  *(124)* | -113  *(790)*  252  *(543)*  -485  *(529)*  -308  *(144)* |
|  |  |  |  |  |  |  |  |  |  |  |  |  |  |

Appendix 4. Mean dwell time of participants (in ms) and standard deviations (in brackets) per Emotion, Block, Condition and Time Point. Participants are spending less time fixating the negative non-social stimuli across the two blocks compared to the social-related emotional stimuli.

|  | **Block 1** | | | | | | | **Block 2** | | | | | | |  |
| --- | --- | --- | --- | --- | --- | --- | --- | --- | --- | --- | --- | --- | --- | --- | --- |
|  | **Social** | | | **Non-Social** | | |  | | **Social** | | | **Non-Social** | | | |
|  | **T1**  **Mean**  **(SD)** | **T2**  **Mean**  **(SD)** | **T3**  **Mean**  **(SD)** | **T1**  **Mean**  **(SD)** | **T2**  **Mean**  **(SD)** | **T3**  **Mean**  **(SD)** |  | | **T1**  **Mean**  **(SD)** | **T2**  **Mean**  **(SD)** | **T3**  **Mean**  **(SD)** | **T1**  **Mean**  **(SD)** | **T2**  **Mean**  **(SD)** | **T3**  **Mean**  **(SD)** | |
|  |  |  |  |  |  |  |  | |  |  |  |  |  |  | |
| **Negative** | 67  *(143)* | 765  *(503)* | 513  *(617)* | 94  *(166)* | 649  *(613)* | 289  *(730)* |  | | -25  *(110)* | 346  *(512)* | 97  *(603)* | 2  *(105)* | 92  *(488)* | -29  *(606)* | |
| **Positive** | 57  *(136)* | 867  *(411)* | 561  *(443)* | 48  *(173)* | 956  *(423)* | 759  *(557)* |  | | -3  *(100)* | 385  *(442)* | 219  *(504)* | 9  *(93)* | 441  *(419)* | 278  *(490)* | |

Appendix 5. 5-HTTLPR genotype groups dwell time (in ms) and standard deviations (in brackets) per Emotion, Block, Condition, and Time Points. Carriers of at least one Short allele are spending less time fixating negative stimuli overall, across blocks, different which is more pronounced for the non-social threat stimuli.

|  | | **Block 1** | | | | | | **Block 2** | | | | | |
| --- | --- | --- | --- | --- | --- | --- | --- | --- | --- | --- | --- | --- | --- |
|  |  | **Social** | | | **Non-Social** | | | **Social** | | | **Non-Social** | | |
|  |  | **T1** | **T2** | **T3** | **T1** | **T2** | **T3** | **T1** | **T2** | **T3** | **T1** | **T2** | **T3** |
|  |  |  |  |  |  |  |  |  |  |  |  |  |  |
| **5-HTTLPR** | | | | | | | |  | | | | | |
| **L/L**  **S/-** | Negative | 80  *(149)* | 905  *(448)* | 719  *(586)* | 129  *(207)* | 945  *(602)* | 617  *(626)* | -7  *(140)* | 368  *(500)* | 117  *(514)* | 8  *(74)* | 296  *(401)* | 178  *(543)* |
|  | Positive | 44  *(163)* | 925  *(398)* | 680 *(403)* | 79  *(228)* | 1065  *(331)* | 877  *(516)* | 23  *(116)* | 501  *(385)* | 274  *(434)* | 35  *(110)* | 479  *(428)* | 225  *(499)* |
|  | Negative | 61  *(141)* | 691  *(521)* | 403  *(614)* | 75  *(140)* | 492  *(567)* | 115  *(730)* | -34  *(92)* | 335  *(526)* | 87  *(653)* | -1  *(118)* | -16  *(501)* | -139  *(616)* |
|  | Positive | 64  *(122)* | 836  *(420)* | 497  *(456)* | 32  *(137)* | 898  *(459)* | 697  *(575)* | -16  *(89)* | 324  *(464)* | 189  *(542)* | -5  *(82)* | 420 *(419)* | 306  *(491)* |
|  |  |  |  |  |  |  |  | |  |  |  |  |  |

Appendix 6. BDNF genotype groups dwell time (in ms) and standard deviations (in brackets) per Emotion, Block, Condition, and Time Points. No significant variations between the two genotypes observed.

|  | | **Block 1** | | | | | | **Block 2** | | | | | |
| --- | --- | --- | --- | --- | --- | --- | --- | --- | --- | --- | --- | --- | --- |
|  |  | **Social** | | | **Non-Social** | | | **Social** | | | **Non-Social** | | |
|  |  | **T1**  **Mean**  **(SD)** | **T2**  **Mean**  **(SD)** | **T3**  **Mean**  **(SD)** | **T1**  **Mean**  **(SD)** | **T2**  **Mean**  **(SD)** | **T3**  **Mean**  **(SD)** | **T1**  **Mean**  **(SD)** | **T2**  **Mean**  **(SD)** | **T3**  **Mean**  **(SD)** | **T1**  **Mean**  **(SD)** | **T2**  **Mean**  **(SD)** | **T3 Mean**  **(SD)** |
|  |  |  |  |  |  |  |  |  |  |  |  |  |  |
| **BDNF Val^66^Met** | | | | | | | |  | | | | | |
| **V/V** | Negative | 83  *(134)* | 825  *(458)* | 527  *(503)* | 132  *(171)* | 669 *(597)* | 295  *(701)* | -40  *(94)* | 398  *(494)* | 149  *(492)* | 5  *(115)* | 181 *(387)* | 86  *(451)* |
|  | Positive | 111  *(111)* | 966  *(433)* | 546  *(481)* | 85  *(160)* | 938  *(427)* | 762  *(617)* | -5  *(101)* | 399  *(466)* | 198  *(525)* | 11  *(95)* | 472  *(330)* | 338  *(438)* |
| **M/-** | Negative | 49  *(154)* | 692  *(555)* | 495  *(747)* | 47  *(150)* | 625  *(645)* | 281  *(781)* | -6  *(127)* | 283  *(539)* | 35  *(723)* | -1  *(93)* | -18  *(580)* | -170  *(741)* |
|  | Positive | -9  *(138)* | 745  *(355)* | 579  *(401)* | 3  *(182)* | 978  *(426)* | 756  *(487)* | 0  *(100)* | 369  *(422)* | 245  *(488)* | 7  *(94)* | 403  *(513)* | 203  *(549)* |

**Appendix 7. Correlation analysis between internalizing and externalizing rates and fixation duration looking the negative and positive stimuli at each Time point and block.**

|  | | **Block 1** | | | | | | | | | | | | **Block 2** | | | | | | | | | | | |  |
| --- | --- | --- | --- | --- | --- | --- | --- | --- | --- | --- | --- | --- | --- | --- | --- | --- | --- | --- | --- | --- | --- | --- | --- | --- | --- | --- |
|  |  | **Social** | | | | | | **Non-Social** | | | | | | **Social** | | | | | | **Non-Social** | | | | | |  |
|  |  | **T1** | | **T2** | | **T3** | | **T1** | | **T2** | | **T3** | | **T1** | | **T2** | | **T3** | | **T1** | | **T2** | | **T3** | |  |
|  |  | |  | |  | |  | |  | |  | |  | |  | |  | |  | |  | |  | |  | |
| **Internalizing** | | | | | | | | | | | | | |  | | | | | | | | | | | |  |
| ***r***  ***p***  ***r***  ***p*** | Negative | | *.192*  *.186* | | *-.167*  *.293* | | *-.071*  *.628* | | *.024*  *.868* | | *.014*  *.922* | | *.058*  *.692* | | *-.088*  *.547* | | *-.080*  *.583* | | *-.041*  *.781* | | *-.100*  *.494* | | *.157*  *.283* | | *.216*  *.137* | |
|  | Positive | | *.083*  *.571* | | *-.070*  *.631* | | *-.025*  *.866* | | *.198*  *.173* | | *.082*  *.576* | | *.056*  *.704* | | *.117*  *.424* | | *-.136*  *.353* | | *-.167*  *.251* | | *.032*  *.830* | | *-.041*  *.780* | | *-.040*  *.786* | |
| **Externalizing**  ***r***  ***p***  ***r***  ***p*** | Negative | | *.335*  *.058* | | *.073*  *.616* | | *.078*  *.594* | | *.076*  *.606* | | *-.074*  *.611* | | *.156*  *.285* | | *-.015*  *.918* | | *.074*  *.612* | | *.159*  *.276* | | *-.126*  *.389* | | *.153*  *.294* | | *.226*  *.119* | |
|  | Positive | | *.108*  *.459* | | *.172*  *.238* | | *.113*  *.439* | | *.211*  *.146* | | *.139*  *.341* | | *.276*  *.055* | | *.240*  *.097* | | *.069*  *.639* | | *-.065*  *.655* | | *.230*  *.112* | | *.139*  *.342* | | *.016*  *.916* | |
